# The PIAS3-Smurf2 sumoylation pathway suppresses breast cancer organoid invasiveness

**DOI:** 10.18632/oncotarget.15471

**Published:** 2017-02-18

**Authors:** Amrita Singh Chandhoke, Ayan Chanda, Kunal Karve, Lili Deng, Shirin Bonni

**Affiliations:** ^1^ Department of Biochemistry and Molecular Biology, and The Arnie Charbonneau Cancer Institute, Cumming School of Medicine, University of Calgary, Calgary, T2N 4N1, Canada

**Keywords:** breast cancer, invasion, sumoylation

## Abstract

Tumor metastasis profoundly reduces the survival of breast cancer patients, but the mechanisms underlying breast cancer invasiveness and metastasis are incompletely understood. Here, we report that the E3 ubiquitin ligase Smurf2 acts in a sumoylation-dependent manner to suppress the invasive behavior of MDA-MB-231 human breast cancer cell-derived organoids. We also find that the SUMO E3 ligase PIAS3 inhibits the invasive growth of breast cancer cell-derived organoids. In mechanistic studies, PIAS3 maintains breast cancer organoids in a non-invasive state via sumoylation of Smurf2. Importantly, the E3 ubiquitin ligase activity is required for sumoylated Smurf2 to suppress the invasive growth of breast cancer-cell derived organoids. Collectively, our findings define a novel role for the PIAS3-Smurf2 sumoylation pathway in the suppression of breast cancer cell invasiveness. These findings lay the foundation for the development of novel biomarkers and targeted therapeutic approaches in breast cancer.

## INTRODUCTION

Breast cancer ranks among the leading causes of mortality in women worldwide. Among the distinct types of breast cancer, basal-like breast cancer displays basal and mesenchymal features [1]. These aggressive tumors lack expression of estrogen receptor (ER), progesterone receptor (PR), and human epidermal growth factor receptor 2 (HER2) are thus referred to as triple-negative breast cancer (TNBC) [2]. Metastasis of these tumors drastically reduce the five-year survival rate of breast cancer patients [3]. Understanding the cellular and molecular mechanisms of metastasis of TNBC has garnered much research interest [4], but these mechanisms remain incompletely understood.

Epithelial-mesenchymal transition (EMT) is thought to play crucial roles in breast cancer invasion and metastasis [5]. EMT comprises loss of apical-basal polarity and cell-cell adhesions resulting from downregulation or mislocalization of epithelial marker proteins such as E-cadherin, and gain of mesenchymal marker proteins [6]. Cells that undergo EMT often display self-renewal capacity, increased motility and metastatic potential [7]. Breast cancer cells may undergo EMT to break away from the local environment within the primary tumor, invade surrounding tissue, transit via the circulatory system and enter new sites where they can establish secondary tumors or metastases [8].

The cytokine Transforming Growth Factor Beta (TGFβ) is a potent inducer of EMT [9]. TGFβ induces mammary epithelial cells to change into mesenchymal cells that migrate and invade tissue. TGFβ is thought to trigger EMT and thereby induce breast cancer cells to invade and metastasize to secondary sites including bone, lung and liver [10, 11].

The ubiquitin E3 ligase Smurf2 suppresses TGFβ-induced EMT in non-transformed mammary epithelial cells [12]. Smurf2 function in the control of EMT is regulated by the SUMO E3 ligase PIAS3, which associates with and triggers Smurf2 sumoylation. Importantly, sumoylation promotes the ability of Smurf2 to induce TGFβ receptor degradation, leading to suppression of TGFβ-Smad-induced EMT [12]. These data have raised the key question of whether the PIAS3-Smurf2 sumoylation pathway might suppress breast cancer invasion and metastasis.

In this study, we report our discovery that the PIAS3-Smurf2 sumoylation pathway suppresses the invasiveness of breast cancer cell-derived organoids. These findings have significant implications for our understanding of breast cancer pathogenesis and management.

## RESULTS

### Smurf2 suppresses the invasive growth of breast cancer cell-derived organoids

Triple negative breast cancer (TNBC) represents 10–20% of human breast cancer [13, 14], but accounts for a significant proportion of breast cancer-related deaths, largely due to the invasive TNBC-derived cells [15]. Therefore, there is an urgent need for better understanding of the mechanisms that control the malignant behavior of TNBC. Epithelial-mesenchymal transition (EMT) has been linked to increased invasion and metastasis in diverse types of cancer including breast carcinomas. Understanding how EMT is regulated in TNBC should facilitate identification of novel approaches of treatment of breast cancer.

The recent identification of Smurf2 as a suppressor of EMT in non-transformed mammary epithelial cells raises the important question of whether Smurf2 might regulate the malignant behavior of breast cancer cells. The three-dimensional culture model system in which cells are grown with extracellular matrix support, such as Matrigel, provides a cellular environment that is thought to simulate the *in vivo* environment. We employed three-dimensional culture as a model system to determine whether Smurf2 regulates the invasiveness of human MDA-MB-231 TNBC cells, which undergo EMT [16, 17]. MDA-MB-231 breast cancer cells form filled spherical multicellular structures with protrusions when grown in three-dimensional Matrigel-supported cultures, mimicking the malignant behavior of breast cancer cells *in vivo* [10, 18]. Further, the cytokine TGFβ induces invasiveness and budding of MDA-MB-231 breast cancer cell-derived organoids [10].

**Figure 1:**
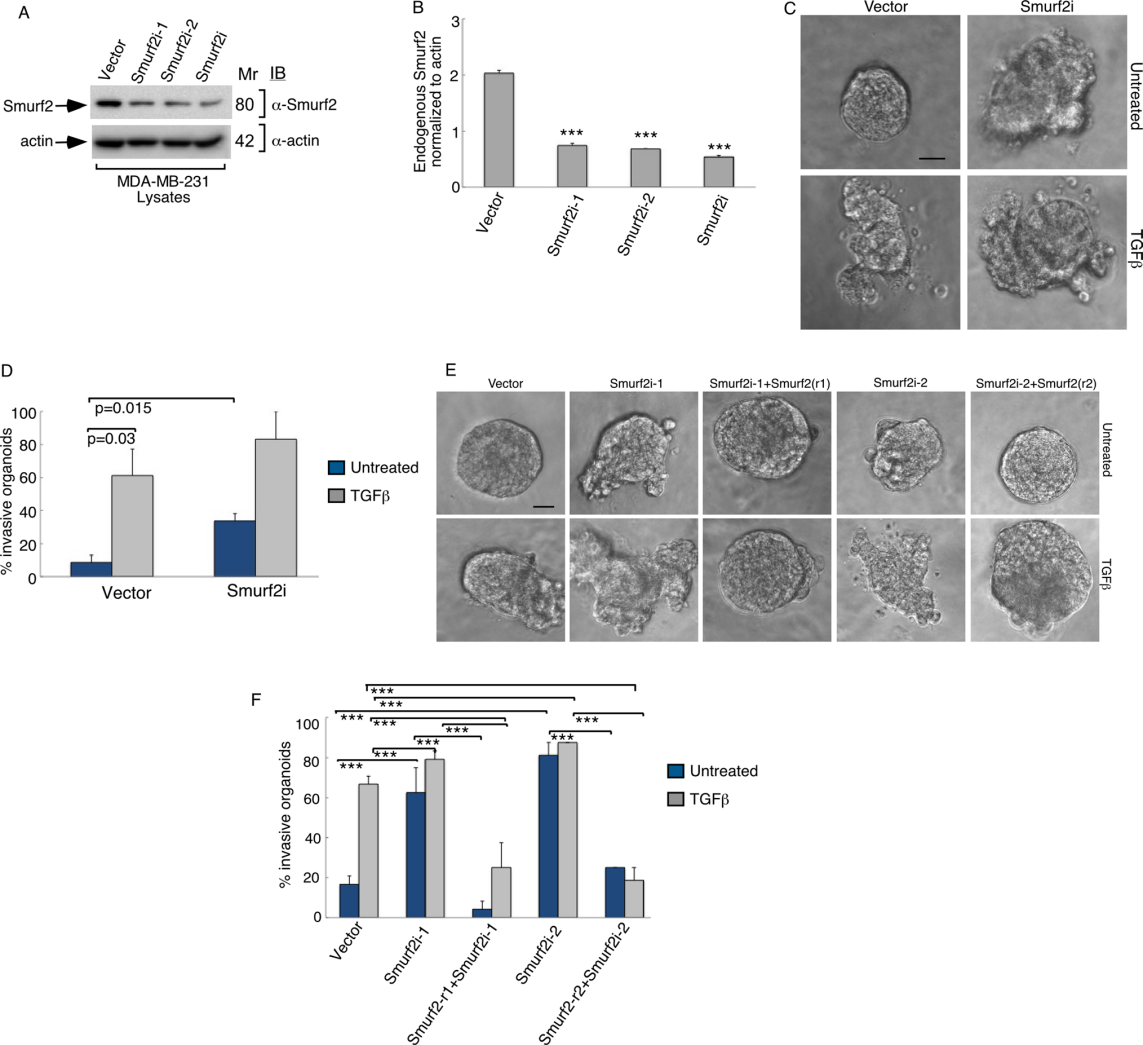
Knockdown of Smurf2 enhances TGFβ-induced disorganization of MDA-MB-231 cancer cell-derived organoids (**A**) Lysates of MDA-MB-231 cells transfected with an RNAi plasmid encoding short hairpin RNAs (shRNAs) targeting one of two unique sequences in Smurf2 (Smurf2i-1 or Smurf2i-2), added separately or as a pool (Smurf2i), or the corresponding U6 RNAi plasmid, were immunoblotted with the Smurf2 or actin antibody, the latter serving as loading control. (**B**) Quantitative analysis of endogenous Smurf2 abundance in lysates of MDA-MB-231 cells transfected as in (A) Bar graph represents the mean ± S.E.M of actin-normalized endogenous Smurf2 abundance expressed relative to the vector control from three independent experiments. Each of the two Smurf2 RNAi plasmids alone or in combination significantly reduced the levels of endogenous Smurf2 in MDA-MB-231 cells. (**C**) Representative micrographs of 10-day live untreated or TGFβ-incubated three-dimensional organoids derived from MDA-MB-231 cells transfected with an RNAi control vector or a pool of Smurf2i-1 and Smurf2i-2 (Smurf2i) plasmids as described in (A). (**D**) Quantification of number of invasive MDA-MB-231 cell-derived organoids transfected and treated as in (C). Bar graph represents the mean ± S.E.M of proportion (%) of invasive organoids from three independent experiments including the one in C. TGFβ promotes the invasive behavior of MDA-MB-231 cell-derived organoids. Knockdown of Smurf2 by RNAi also promoted the invasive organoid growth even in the absence of TGFβ. (**E**) Representative micrographs of 10-day live untreated or TGFβ-incubated organoids derived from MDA-MB-231 cells transfected with control vector alone, or Smurf2i-1 and Smurf2i-2, together with the plasmid expressing Smurf2-r1 and Smurf2-r2, respectively, or with a mammalian expression vector control. (**F**) Quantification of number of invasive MDA-MB-231 cell-derived organoids transfected and treated as in (E) Bar graph represents the mean ± S.E.M of proportion (%) of invasive organoids from three independent experiments including the one in (E) Smurf2-r1and Smurf2-r2, respectively, reversed Smurf2i-1 and Smurf2i-2-induced invasive behavior of MDA-MB-231 cell-derived organoids in absence and presence of TGFβ. In addition, Smurf2-r1 or Smurf2-r2 coexpression with Smurf2 RNAi suppressed the ability of TGFβ to induce invasive behavior of organoids relative to vector control cells. Significant difference, ANOVA: ***p ≤ 0.001. Scale bar = 50 μm. Mr refers to relative Molecular Mass.

We first assessed the role of endogenous Smurf2 in regulating the invasive growth of three-dimensional MDA-MB-231 breast cancer cell-derived organoids using RNA interference (RNAi). We employed two short hairpin RNAs (shRNAs) targeting distinct sequences within Smurf2, which individually or in combination led to efficient knockdown of endogenous Smurf2 in MDA-MB-231 breast cancer cells (Figure 1A, 1B) [12]. As expected, addition of TGFβ induced the deformation of three-dimensional MDA-MB-231 breast cancer cell-derived organoids, as indicated by the loss of spherical structures and appearance of outward protrusions (Figure 1C). Interestingly, knockdown of Smurf2 by RNAi induced outward growth, budding and branching of MDA-MB-231 cell-derived organoids even in the absence of TGFβ (Figure 1C). Quantitative analyses of the relative number of organoids showing an invasive behavior confirmed that TGFβ promoted invasive growth of MDA-MB-231 cell-derived organoids (Figure 1D). Importantly, Smurf2 shRNAs increased the proportion of invasive breast cancer cell-derived organoids, even in the absence of TGFβ (Figure 1D). Together, these data suggest that endogenous Smurf2 suppresses the aggressive behavior of breast cancer-derived organoids.

**Figure 2:**
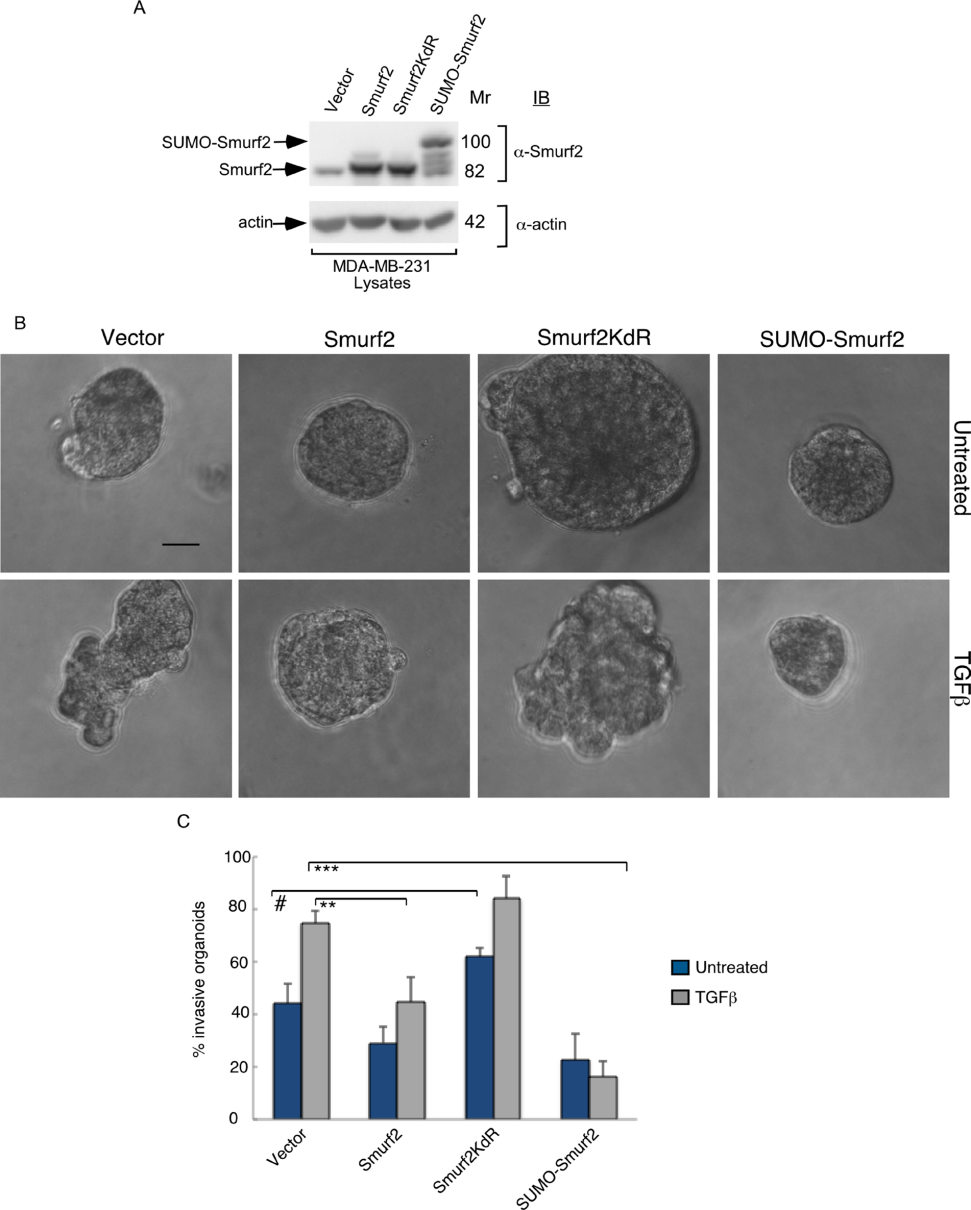
Smurf2 suppresses TGFβ–induced invasive growth of MDA-MB-231 cell-derived organoids in a sumoylation-dependent manner (**A**) Lysates of MDA-MB-231 cells expressing wild type Smurf2, Smurf2KdR, or SUMO-Smurf2, or transfected with the control vector, were immunoblotted with the Smurf2 or actin antibody, the latter serving as loading control. (**B**) Representative micrographs of 10-day live untreated or TGFβ-incubated three-dimensional organoids derived from different MDA-MB-231 cells as in (A). (**C**) Quantification of number of invasive MDA-MB-231 cell-derived organoids transfected and treated as in A. Bar graph represents the mean ± S.E.M of the proportion (%) of invasive organoids from three independent experiments including the one in (A) Smurf2 and SUMO-Smurf2 suppressed the ability of TGFβ to induce invasive growth in MDA-MB-231 cells. In contrast, Smurf2KdR promoted the invasive growth of breast cancer cell-derived organoids even in the absence of TGFβ. Significant difference, ANOVA: **p ≤ 0.01, ***p ≤ 0.001. Student *t*-test: ^#^*p* ≤ 0.05. Scale bar = 50 μm. Mr refers to relative Molecular Mass.

To exclude the possibility of off-target effects of Smurf2 RNAi on the invasive growth behavior of MDA-MB-231 cell-derived organoids, we performed a rescue experiment. Coexpression of shRNAi-1-resistant Smurf2 (Smurf2-r1) or shRNAi-2-resistant Smurf2 (Smurf2-r2) reversed the invasive growth of MDA-MB-231 cell-derived organoids induced by Smurf2-1 shRNAs and Smurf2-2 shRNAs, respectively (Figure 1E, 1F). Expression of Smurf2-r1 or Smurf2-r2 in the background of Smurf2 RNAi with the cognate shRNA also suppressed the ability of TGFβ to promote invasive growth of MDA-MB-231-derived organoids (Figure 1E, 1F). In complementary studies, expression of Smurf2 suppressed the ability of TGFβ to promote the invasive growth of three-dimensional breast cancer-cell derived organoids (Figure 2). Collectively, these findings suggest that Smurf2 inhibits invasiveness and deformation of three-dimensional-breast cancer cell-derived multicellular structures.

### Smurf2 suppresses the invasive growth of MDA-MB-231 cell-derived organoids in a sumoylation-dependent manner

The findings that Smurf2 suppresses the invasive growth of breast cancer cell-derived multicellular structures raised the key question of how Smurf2 might be regulated. Smurf2 is a target of the SUMO pathway, where the SUMO E3 ligase PIAS3 triggers the sumoylation of Smurf2 at Lysines 26 and 369 [12]. Sumoylation promotes the ability of Smurf2 to suppress EMT in non-transformed mammary epithelial cells [12]. We, therefore, measured the effect of sumoylation on the ability of Smurf2 to suppress the invasive growth of MDA-MB-231 breast cell-derived organoids. Smurf2KdR, in which Lysines 26 and 369 are replaced with arginine serves as a SUMO loss of function Smurf2, and the SUMO-Smurf2 fusion protein, in which a SUMO isopeptidase-resistant SUMO protein is N-terminally fused to Smurf2 represents SUMO gain of function of Smurf2 (Figure 2A). We found that expression of SUMO-Smurf2 or wild type Smurf2 suppressed TGFβ-induced invasive growth of three-dimensional MDA-MB-231 cell-derived organoids (Figure 2B, 2C). However, expression of Smurf2KdR increased the invasive behavior of breast cancer cell-derived organoids even in the absence of TGFβ (Figure 2B, 2C), suggesting that Smurf2KdR may act in a dominant interfering fashion in breast cancer cells. Together, these data suggest that the sumoylation of Smurf2 suppresses the invasiveness of three-dimensional breast cell-derived multicellular structures.

### PIAS3 suppresses the invasive growth of MDA-MB-231 cell-derived organoids via sumoylated Smurf2

**Figure 3:**
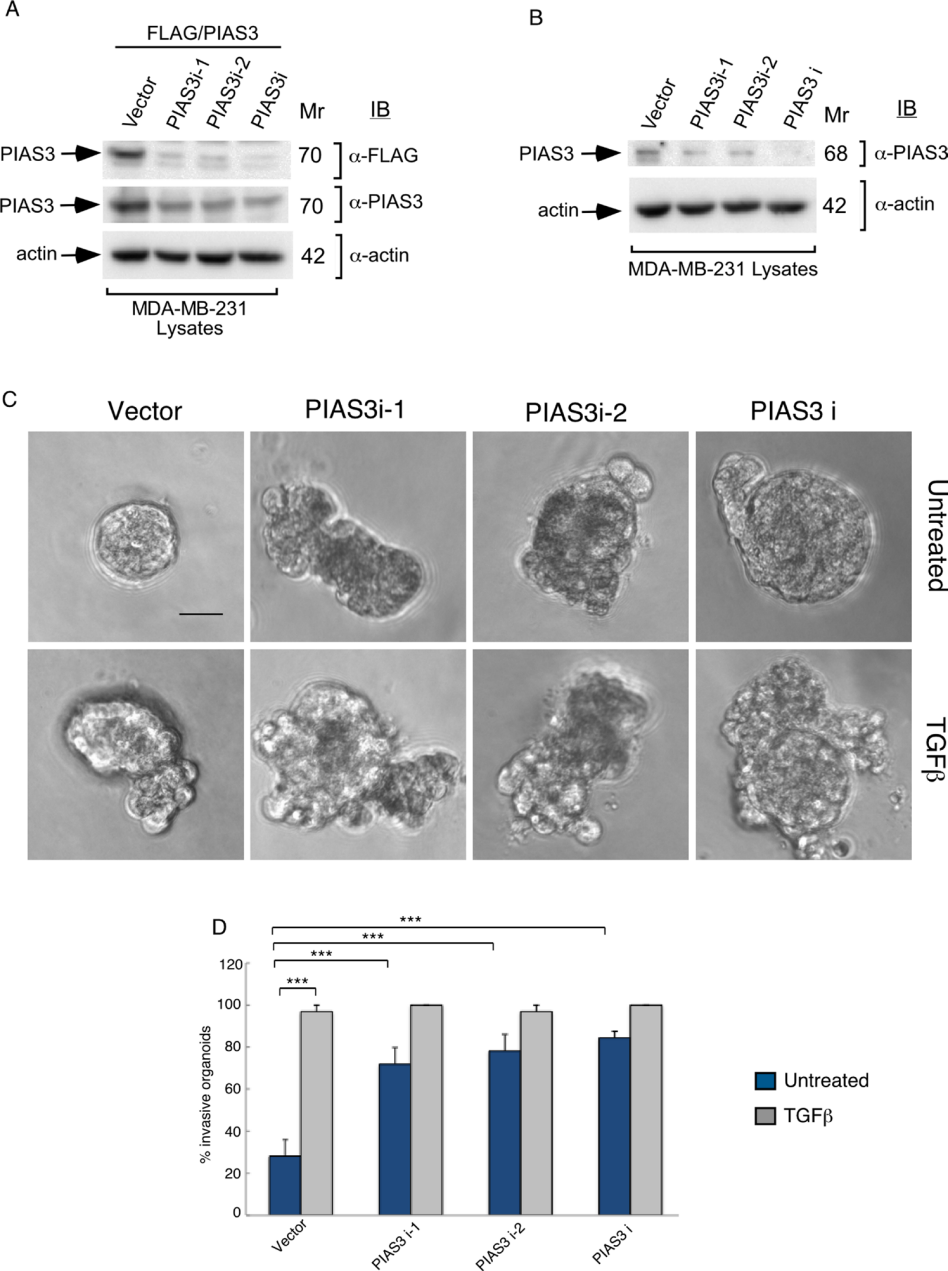
Knockdown of PIAS3 disrupts MDA-MB-231 breast cancer cell-derived organoids (**A**) FLAG, PIAS3 or actin immunoblots of lysates of MDA-MB-231 cells cotransfected with a plasmid encoding FLAG-tagged PIAS3 protein, together with an RNAi plasmid encoding short hairpin RNAs (shRNAs) targeting one of two unique sequences in PIAS3, alone or together, or the corresponding U6 RNAi plasmid. Actin served as loading control. The immunoblots are from an experiment that was repeated three independent times with similar results. Expression of PIAS3 shRNA-1 (PIAS3i-1), PIAS3 shRNA-2 (PIAS3i-2) alone or together reduced the abundance of exogenous PIAS3 by more than 90%. (**B**) PIAS3 and actin immunoblots of lysates of MDA-MB-231 cells transfected with RNAi plasmids encoding short hairpin RNAs (shRNAs) targeting one of two unique sequences in PIAS3 (PIAS3i-1 or PIAS3i-2), added separately or together, or transfected with U6 RNAi plasmid. Actin served as loading control. The immunoblots are from an experiment that was repeated two independent times with similar results. Expression of PIAS3 shRNA-1 (PIAS3i-1), PIAS3 shRNA-2 (PIAS3i-2) alone or together reduced the abundance of endogenous PIAS3 by more than 60%. (**C**) Representative micrographs of 10-day-old live untreated or TGFβ-incubated three-dimensional organoids derived from MDA-MB-231 cells transfected with an RNAi control vector, PIAS3i-1 plasmid, PIAS3i-2 plasmid or a pool of PIAS3i-1 and PIAS3i-2 (PIAS3i) plasmids as described in (B). (**D**) Quantification of number of invasive MDA-MB-231 cell-derived organoids transfected and treated as in C. Bar graph represents the mean ± S.E.M of proportion (%) of invasive organoids from four independent experiments including the one in (D). Endogenous PIAS3 knockdown by PIAS3 shRNA-1, PIAS3 shRNA-2, alone or together promoted invasive growth of MDA-MB-231-derived organoids. Significant difference, ANOVA: ***p ≤ 0.001. Scale bar = 50 μm. Mr refers to relative Molecular Mass.

**Figure 4:**
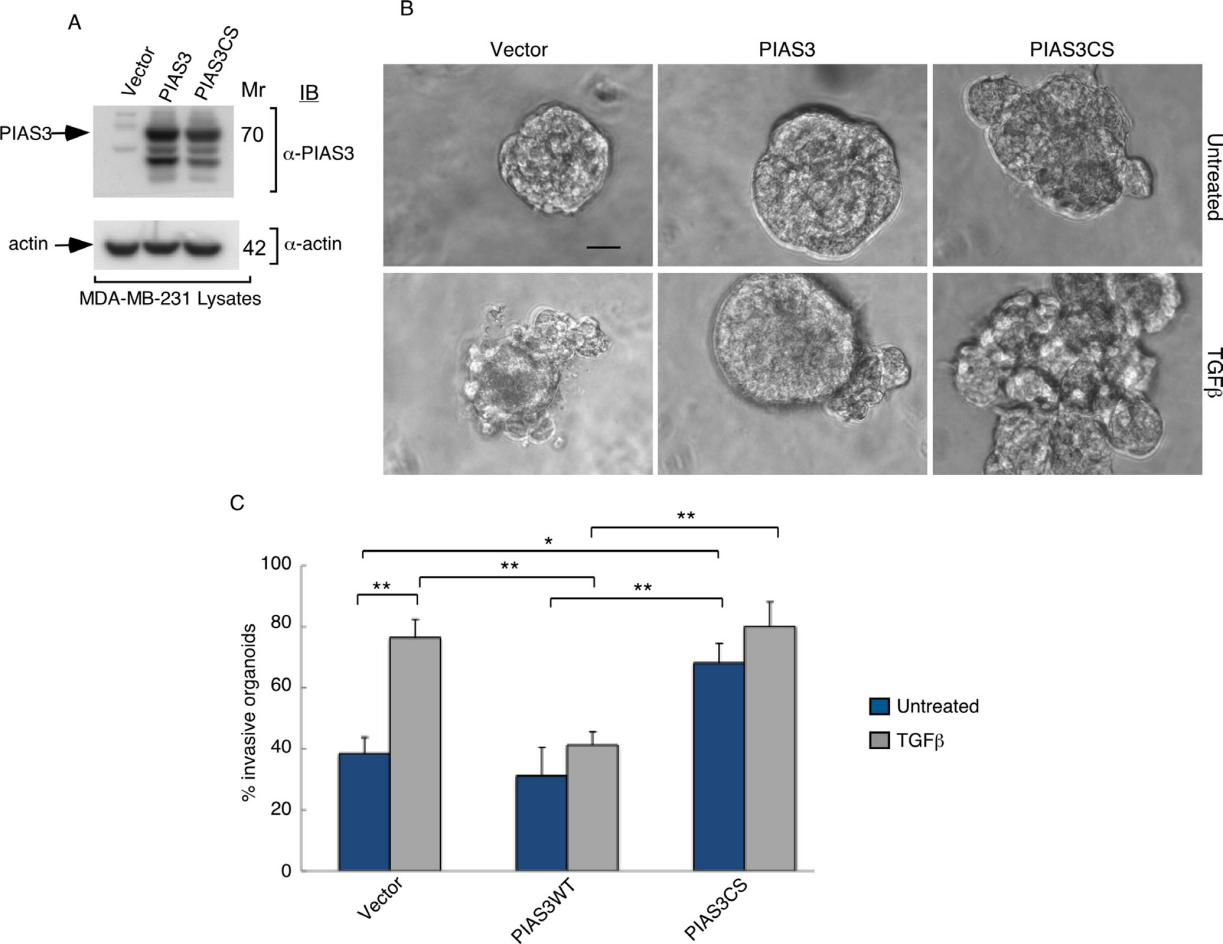
PIAS3 suppresses TGFβ-induced invasive growth of MDA-MB-231 breast cancer cell-derived organoids in a SUMO E3 ligase activity-dependent manner (**A**) PIAS3 and actin immunoblots of lysate of MDA-MB-231 cells expressing FLAG-tagged PIAS3 or the SUMO E3 ligase inactive PIAS3CS, or transfected with the control vector. Actin served as a loading control. (**B**) Representative micrographs of 10-day live untreated or TGFβ-incubated three-dimensional organoids derived from MDA-MB-231 cells transfected as in (A). (**C**) Quantification of number of invasive MDA-MB-231 cell-derived organoids transfected and treated as in A. Bar graph represents the mean ± S.E.M of proportion (%) of invasive organoids from seven independent experiments including the one in A. PIAS3 significantly suppressed the ability of TGFβ to induce invasive growth in MDA-MB-231 cell-derived organoids. Interestingly, PIAS3CS promoted the invasive growth of MDA-MB-231 cell-derived organoids even in the absence of TGFβ addition. Significant difference, ANOVA: **p ≤ 0.01, *p ≤ 0.05. Scale bar = 50 μm. Mr refers to relative Molecular Mass.

The SUMO E3 ligase PIAS3 binds and promotes Smurf2 sumoylation. We asked if PIAS3 might regulate the invasive behavior of MDA-MB-231 cell-derived organoids. We, first, measured the effect of knockdown of endogenous PIAS3 on the invasive growth of MDA-MB-231 cell-derived multicellular structures. We confirmed that expression of PIAS3i-1 and PIAS3i-2 shRNAs targeting two distinct regions within PIAS3 induced knockdown of exogenous as well as endogenous PIAS3 in MDA-MB-231 cells (Figure 3A and 3B). In three-dimensional assays MDA-MB-231 cell-derived organoids formed filled spheres, and TGFβ led to their deformation and invasiveness (Figure 3C). However, knockdown by either one or both PIAS3 shRNAs triggered outward growth, budding and branching of three-dimensional MDA-MB-231 cell-derived organoids even in the absence of TGFβ (Figure 3C). Quantitative analyses of the proportion of MDA-MB-231 breast cancer cell-derived organoids showing an invasive behavior revealed that expression of PIAS3 shRNA-1 and PIAS3 shRNA-2 alone or together led to 65% increase in the number of breast cancer cell-derived organoids with invasive growth (Figure 3D). In complementary experiments, expression of PIAS3 promoted the appearance of organized MDA-MB-231 cell-derived multicellular spherical structures and reduced the proportion of organoids with protrusions (Figure 4A–4C). Remarkably, expression of the SUMO E3 ligase inactive PIAS3CS, in which the RING finger Cysteine 299 is converted to serine, promoted the invasive growth of breast cancer cell-derived organoids even in the absence of TGFβ (Figure 4A–4C) phenocopying the effect of PIAS3 knockdown (Figure 3C, 3D). Together, these findings suggest that endogenous PIAS3 suppresses the aggressive behavior of breast cancer-derived organoids in a SUMO E3 ligase-dependent manner.

**Figure 5:**
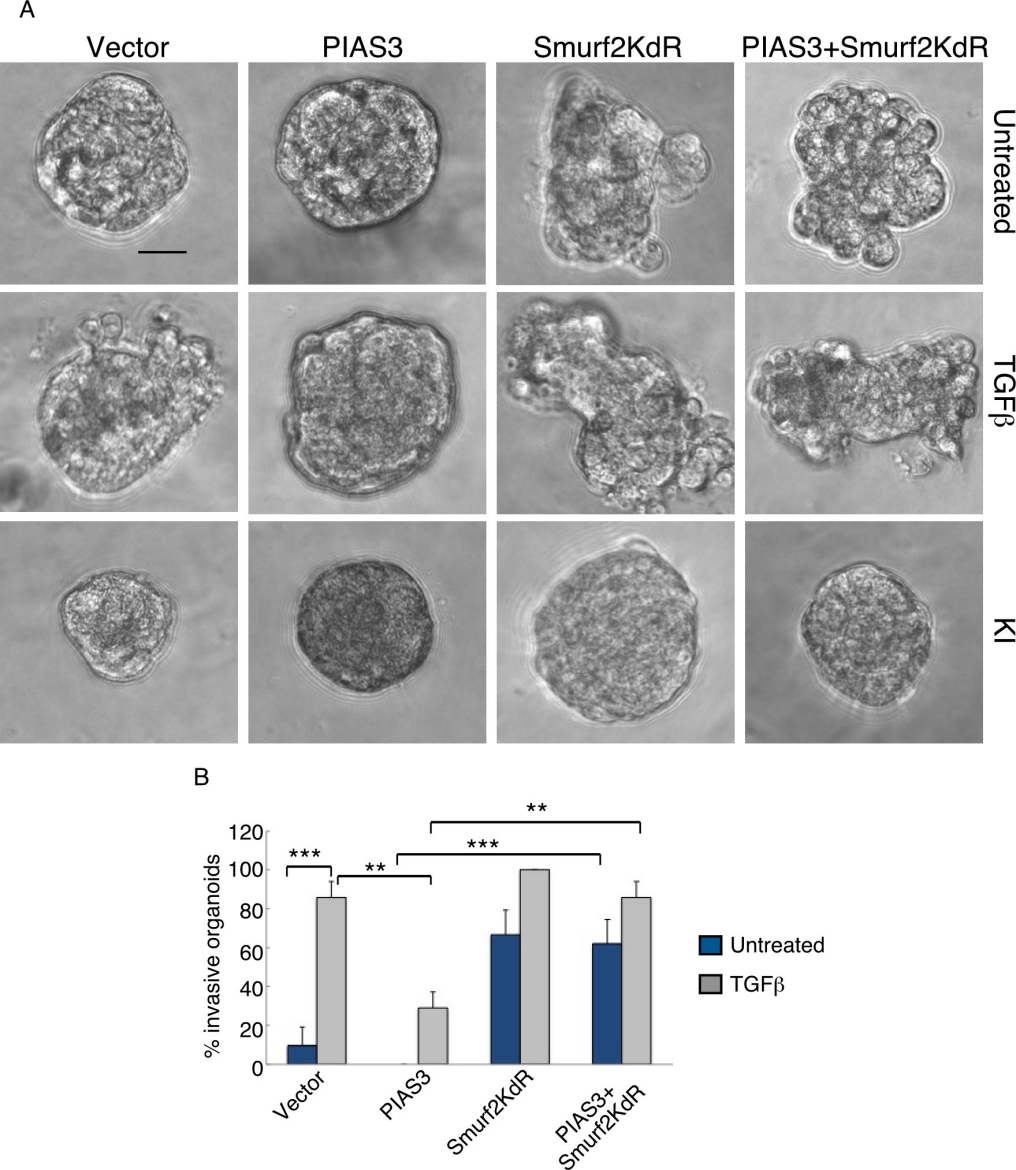
Sumoylation-defective Smurf2 blocks PIAS3 suppression of invasive growth of MDA-MB-231 breast cancer cell-derived organoids (**A**) Representative DIC images of 10-day-old live untreated, TGFβ-treated, or KI-treated three-dimensional multicellular structures from MDA-MB-231 cells expressing wild type PIAS3, the SUMO loss of function Smurf2KdR, alone or together, or transected with the vector control. KI refers to the TGFβ type I receptor kinase inhibitor SB431542. (**B**) Quantification of number of invasive untreated or TGFβ -treated MDA-MB-231 cell-derived organoids transfected as in A. Bar graph represents the mean ± S.E.M. of the proportion (%) of invasive organoids from three independent experiments including the one in A. Smurf2KdR reversed the ability of PIAS3 to suppress TGFβ-induced invasive growth of MDA-MB-231 cell-derived organoids in absence and presence of TGFβ. Smurf2KdR-induced invasive growth of MDA-MB-231 cell-derived organoids is reversed by incubation of the three-dimensional cultures with 10 μM KI. Significant difference, ANOVA: **p ≤ 0.01 and ***p ≤ 0.001. Scale bar = 50 μm.

**Figure 6:**
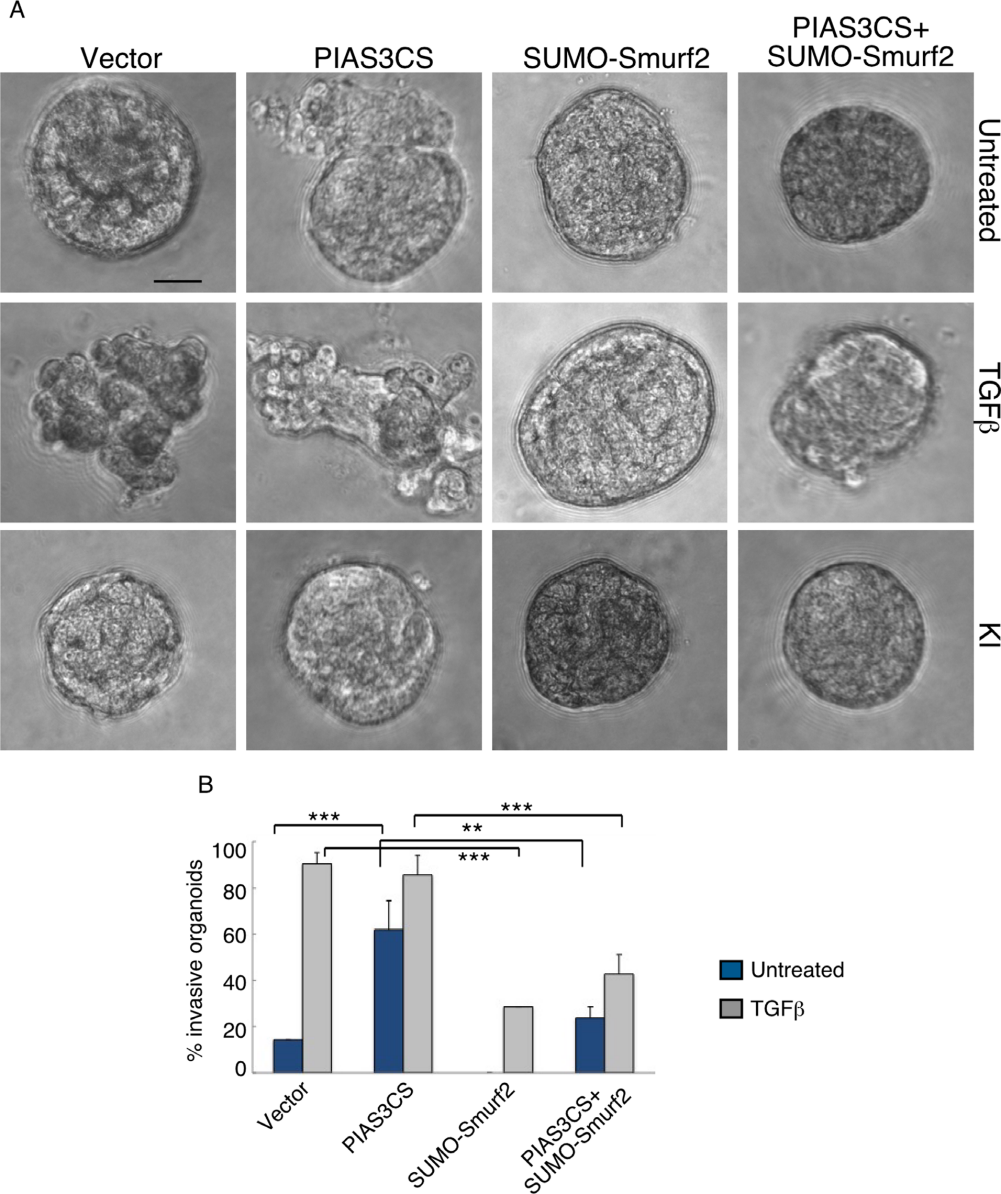
SUMO-Smurf2 reverses PIAS3CS to induce invasive growth of MDA-MB-231 breast cancer cell-derived organoids (**A**) Representative DIC images of 10-day-old live untreated, TGFβ-treated, or KI-treated three-dimensional multicellular structures from MDA-MB-231 cells expressing the SUMO E3 ligase inactive PIAS3CS, the SUMO gain-of-function SUMO-Smurf2, alone or together, or transfected with the vector control. KI refers to the TGFβ type I receptor kinase inhibitor SB431542. (**B**) Quantification of invasive MDA-MB-231 cell-derived organoids transfected as in A and either left untreated or incubated with TGFβ. Bar graph represents the mean ± S.E.M. of the proportion (%) of invasive organoids from three independent experiments including the one in (**A**). SUMO-Smurf2 reversed the ability of PIAS3CS to promote TGFβ-induced invasive growth of MDA-MB-231 cell-derived spheroids both in absence and presence of TGFβ. PIAS3CS-induced invasive growth of MDA-MB-231 cell-derived organoids is reversed by incubation of the three-dimensional cultures with 10 μM of KI. Significant difference, ANOVA: **p ≤ 0.01 and ***p ≤ 0.001. Scale bar = 50 μm

We next determined the relationship between PIAS3 and Smurf2 in the control of the malignant behavior of breast cancer cells. We found that expression of Smurf2KdR blocked the ability of PIAS3 to suppress the invasive behavior of MDA-MB-231 cell-derived organoids in the presence of TGFβ (Figure 5A, 5B). In complementary analyses, expression of SUMO-Smurf2 suppressed the ability of PIAS3CS to induce invasive growth of MDA-MB-231 cell-derived organoids (Figure 6A, 6B). Incubation of three-dimensional cultures with a TGFβ type I receptor kinase inhibitor (KI) reversed the ability Smurf2KdR and PIAS3CS to induce invasive growth of MDA-MB-231 breast cancer-derived organoids, suggesting that Smurf2KdR and PIAS3CS promote invasiveness of breast cancer cells via basal TGFβ signaling (Figure 5A, 6A: third row). Together, these data suggest that PIAS3 acts via sumoylation of Smurf2 to inhibit the invasive behavior of breast cancer cell-derived organoids.

### E3 ubiquitin ligase activity is required for sumoylated Smurf2 to suppress invasive growth of MDA-MB-231 cell-derived organoids

**Figure 7:**
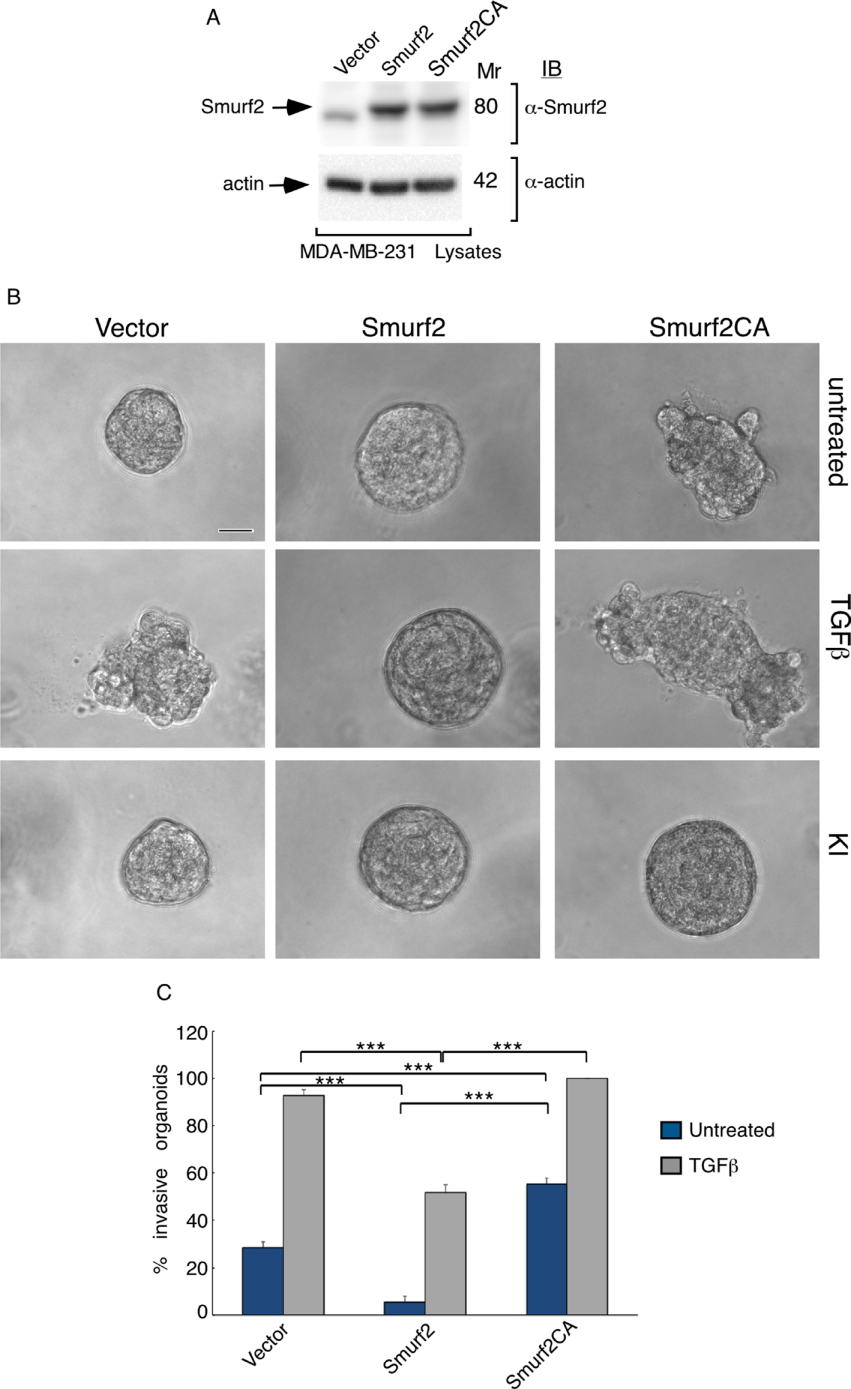
E3 ubiquitin ligase activity is required for Smurf2 to suppress TGFβ-induced invasive growth of MDA-MB-231 cell-derived organoids (**A**) Lysates of MDA-MB-231 cells expressing wild type Smurf2 or the ubiquitin E3 ligase inactive Smurf2CA in which Cysteine 716 is mutated to alanine, or transfected with the control vector, were immunoblotted with the MYC or actin antibody, the latter serving as loading control. (**B**) Representative micrographs of 10-day live untreated, TGFβ incubated or KI-incubated three-dimensional organoids derived from different MDA-MB-231 cells as in (A). (**C**) Quantification of number of invasive MDA-MB-231 cell-derived organoids transfected and left untreated or incubated with TGFβ are shown. Bar graph represents the mean ± S.E.M of the proportion (%) of invasive organoids from seven independent experiments including the one in (A). Smurf2 suppressed the ability of TGFβ to induce invasive growth in MDA-MB-231 cell-derived organoids. In contrast, Smurf2CA promoted the invasive growth of breast cancer cell-derived organoids even in the absence of TGFβ. Significant difference, ANOVA: ***p ≤ 0.001. Scale bar = 50 μm. Mr refers to relative Molecular Mass.

In light of the observation that Smurf2 targets the TGFβ receptors for ubiquitin-mediated degradation to suppress TGFβ signaling and biological effects [19, 20], our findings raised the question of whether the E3 ubiquitin ligase activity is required for the ability of Smurf2 to suppress TGFβ-induced invasive growth of breast cancer-derived organoids. Therefore, we compared the effect of expression of wild type Smurf2 and the ubiquitin E3 ligase inactive Smurf2CA, in which the catalytic Cysteine residue 716 was converted to serine, on MDA-MB-231-cell-derived organoids. Expression of wild type Smurf2 suppressed the ability of TGFβ to induce invasive growth of MDA-MB-231 cell-derived organoids (Figure 7). By contrast, expression of Smuf2CA promoted the invasive growth of MDA-MB-231 cell-derived organoids in the absence or presence TGFβ, suggesting that the ubiquitin E3 ligase activity is critical for Smurf2 to suppress breast cancer-organoid invasive behavior (Figure 7). These data suggest that the ubiquitin E3 ligase activity is required for Smurf2 to suppress the invasive behavior of breast cancer cell-derived organoids (Figures 1, 2, 5 and 7).

**Figure 8:**
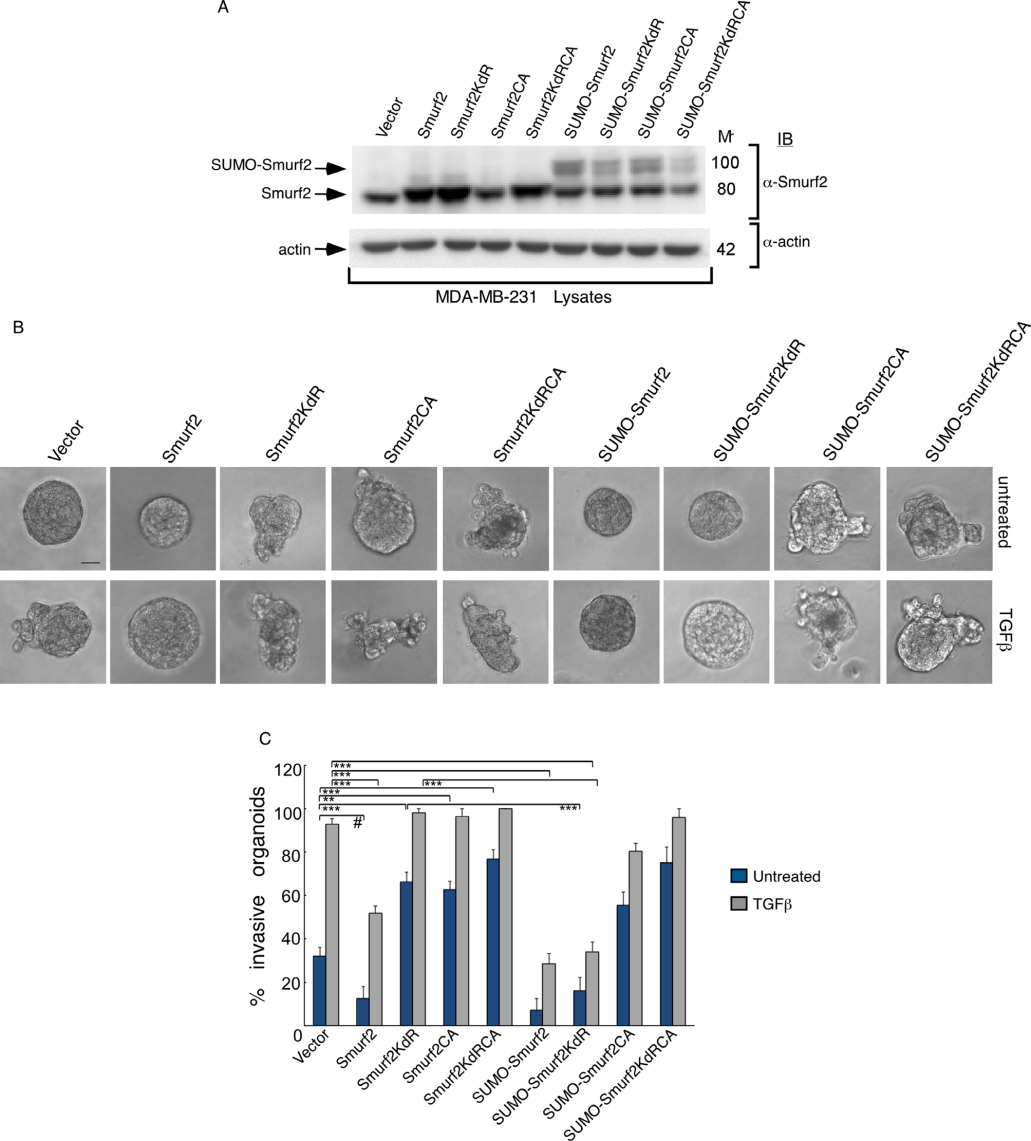
Sumoylated Smurf2 requires an intact E3 ubiquitin ligase activity to suppress TGFβ-induced invasive growth of MDA-MB-231 cell-derived organoids (**A**) Lysates of MDA-MB-231 cells expressing wild type Smurf2, Smurf2KdR, Smurf2CA, SmurfKdRCA, SUMO-Smurf2, SUMO-Smurf2KdR, SUMO-Smurf2CA, SUMO-Smurf2KdRCA, or transfected with the control vector, were immunoblotted with the Smurf2 or actin antibody, the latter serving as loading control. (**B**) Representative micrographs of 10-day live untreated or TGFβ-incubated three-dimensional organoids derived from MDA-MB-231 cells transfected as in (A). (**C**) Quantification of number of invasive MDA-MB-231 cell-derived organoids transfected and treated as in A. Bar graph represents the mean ± S.E.M of the proportion (%) of invasive organoids (*n* = 7 experiments for all except for SUMO-Smurf2KdRCA, which were repeated three times) including the one in A. SUMO-Smurf2KdR suppressed, whereas SUMO-Smurf2CA and SUMO-Smurf2KdRCA promoted the invasive growth of breast cancer cell-derived organoids even in the absence of TGFβ. Significant difference, ANOVA: ***p ≤ 0.001. Student *t*-test: ^#^ *p* ≤ 0.001. Scale bar = 50 μm. Mr refers to relative Molecular Mass.

These data also raised the key question whether sumoylation and ubiquitin E3 ligase activity of Smurf2 are functionally linked. Interestingly, sumoylation promotes the ability of Smurf2 to reduce the abundance of TGFβ receptors leading to suppression of TGFβ-induced EMT in three-dimensional non-transformed mammary epithelial cell-derived organoids [12]. To further characterize the relationship between sumoylation and E3 ubiquitin ligase activity of Smurf2 in its function in the control of tumor invasiveness, we determined the effect of fusion of SUMO on the ability of Smurf2KdR, Smurf2CA, or Smurf2KdRCA to promote the invasive growth of the three-dimensional-breast cancer organoids. Remarkably, we found that expression of SUMO-Smurf2KdR rescued the non-invasive growth of three-dimensional MDA-MB-231 organoids (Figure 8). In contrast, expression of SUMO-Smurf2CA or SUMO-Smurf2KdRCA failed to suppress the invasive growth of these organoids (Figure 8). Collectively, our data suggest that the E3 ubiquitin ligase activity of Smurf2 is required for sumoylated Smurf2 to suppress the invasive growth of breast cancer-derived organoids.

## DISCUSSION

In this study, we have uncovered an important role for sumoylation in the ability of the ubiquitin E3 ligase Smurf2 to suppress the invasive behavior of breast cancer cell-derived organoids. We have also identified a novel role for the SUMO E3 ligase PIAS3 in maintaining a non-invasive phenotype of MDA-MB-231 cell-derived organoids. Mechanistically, our data suggest that PIAS3 acts via sumoylation of Smurf2 to suppress the invasive growth of breast cancer cells. In addition, our data support a crucial role for the ubiquitin E3 ligase activity of Smurf2 in mediating the ability of sumoylated Smurf2 to suppress the invasive growth of breast cancer-derived organoids. These findings define a key role for the PIAS3-Smurf2 sumoylation pathway in the suppression of the malignant behavior of cancer cells.

Our findings illuminate the roles and mechanisms of Smurf2 in the control of breast cancer pathogenesis. Smurf2 may have complex roles in tumorigenesis [21–23]. Consistent with our findings, Smurf2 knockout mice show spontaneous tumor formation including in mammary glands [24], suggesting a tumor suppressor role for Smurf2 in breast cancer. Consistent with these results, the abundance of Smurf2 protein is reduced in breast cancer [21, 24, 25]. Notably, miR-15b family members may downregulate Smurf2 levels, a phenomenon that appears to be associated with breast cancer metastasis [21]. By contrast, Smurf2 has also been suggested to promote the proliferation of breast cancer tumor cells, tumor formation and metastasis in xenograft models [22]. Our study suggests that sumoylation may control Smurf2 function in breast cancer invasion and metastasis. In future studies, it will be important to determine the role of Smurf2 sumoylation in breast cancer metastasis in animal models.

Our findings also highlight a role for PIAS3 in EMT and cancer invasion and metastasis. The role of PIAS3 in breast cancer remains to be elucidated [26–28]. The related protein PIAS1 acts in a SUMO E3 ligase-dependent manner to suppress EMT in mammary epithelial cells as well as the invasive growth or metastatic growth of breast cancer cells in three-dimensional cultures and xenograft model [10, 29]. As our data in the current study suggest that PIAS3 acts via sumoylation of Smurf2 to suppress the invasive behavior of breast cancer cell-derived organoids, it will be important in future studies to determine the role of PIAS3-Smurf2 sumoylation pathway in the metastatic potential of human breast cancer.

It will be interesting to probe whether the protein abundance or activity of PIAS3 might be altered in breast cancer. Our data support the idea that reduction in the abundance of PIAS3 might be important for the invasive behavior of breast cancer cell-derived organoids. These data also suggest that PIAS3 and Smurf2 may serve as biologically relevant biomarkers in human breast cancer.

In conclusion, our findings demonstrate that the PIAS3-Smurf2 sumoylation pathway suppresses the invasive behavior of breast cancer cell-derived organoids. Future studies investigating the relevance of PIAS3-Smurf2 sumoylation pathway in breast cancer invasion and metastasis will help uncover novel biomarkers and therapeutic targets for breast cancer.

## MATERIALS AND METHODS

### Plasmids

CMV-based mammalian expression plasmids containing cDNA encoding MYC-tagged Smurf2 (MYC-Smurf2), Smurf2 in which Cysteine 716 is converted to alanine (Smurf2CA), Smurf2 in which Lysines 26 and 369 are converted to arginine (MYC-Smurf2KdR), and SUMO-Smurf2 proteins have been previously described [12, 30–32]. MYC-tagged Smurf2KdRCA, SUMO-Smurf2KdR, SUMO-Smurf2CA and SUMO-Smuf2KdRCA were generated using restriction-based subcloning strategies. The FLAG-tagged wild type PIAS3 and the SUMO E3 ligase inactive PIAS3CS, in which the Ring domain Cysteine 299 is converted to serine, were kind gifts from Dr. Kulesz Martin (Oregon Health and Science University) [33]. U6-based Smurf2 RNAi vectors encoding Smurf2 hairpin RNA (shRNA)1 and Smurf2 shRNA2 targeting two distinct regions in Smurf2 ((5′-GGGCCAAATGACAATGA TACA-3′-Smurf2i-1) and (5′-GGGAAGTCAATTACCTTGGAT-3′-Smurf2i-2)) to knockdown Smurf2 are as previously described [12, 34, 35]. Smurf2 rescue constructs, which are RNAi-resistant Smurf2 plasmids, Smurf2(r1) and Smurf2(r2) are generated as described previously [12, 35, 36]. U6-based PIAS3 RNAi vectors encoding PIAS3 shRNA1 and PIAS3 shRNA2 targeting two distinct regions in PIAS3 ((5′-AAGCTCATCAGATGAGGAGGAT-3′ -PIAS3i-1) and 5′GAAGTCTA TGGGGAGCTCATCCG-3′ -PIAS3i-2) to knockdown PIAS3 are generated as described previously [12, 35, 36]. The sequences of all plasmids were confirmed by DNA sequence analysis at University of Calgary Core Sequencing Facility, AB, Canada.

### Cell lines and transfection

Human MDA-MB-231 breast cancer cells and human epithelial kidney (HEK) 293T cells were cultured in Dulbecco's modified Eagle's medium with high glucose and L-glutamine supplemented with 10% fetal bovine serum (Invitrogen, Canada). MDA-MB-231 cells were transfected using Lipofectamine LTX reagent (Invitrogen, Canada). 293T cells were transfected using the Calcium phosphate precipitation method. Cells were confirmed to be free of pathogenic Mycoplasma strain using PCR-ELISA kit.

### Cell extract preparation and Immunoblotting

Cells were lysed in TNTE lysis buffer (50 mM Tris, 150 mM NaCl, 1 mM EDTA, 0.5% [v/v] Triton-X 100) containing protease inhibitors and phosphate inhibitors as described [34, 35, 37]. Lysates were centrifuged at 13000 × g for 10 minutes at 4°C. Supernatants of cell lysates were collected, small aliquots of which were subjected to protein concentration determination using Bradford-based protein assays. Cell extracts were resolved by SDS-PAGE, followed by transfer to nitrocellulose membranes. The blots were then incubated with specific primary antibodies including mouse anti-PIAS3 (Santa Cruz, sc46682), mouse anti-actin (Santa Cruz, sc47778), mouse anti-Smurf2 (Santa Cruz, sc393848) followed by HRP-conjugated goat anti-mouse as secondary antibodies (Jackson Laboratories). Blots were then incubated with ECL (EMD Millipore Canada) and signals were detected using a VersaDoc 5000 Imager (Bio-Rad Laboratories). Densitometry was performed using Quantity One software (Bio-Rad Laboratories).

### Three-dimensional breast cancer cell-derived organoid preparation

Transiently transfected MDA-MB-231 breast cancer cells, were trypsinised and prepared for three-dimensional culture as described elsewhere [10, 12, 38]. 96-well flat-bottom ultra-low attachment plates were coated with 50 μL of 30% diluted growth-factor-reduced Matrigel (3 mg/ml) (BD bioscience) in complete growth medium containing antibiotics and antimitotic agents [10, 12, 38]. The Matrigel-coated 96-well plates were kept at 37°C in a 5% CO_2_ incubator for 1 hour to allow the Matrigel cushions to set. 50 μL of 30% Matrigel-MDA-MB-231 cell suspension was carefully layered on top of the Matrigel bed. On the next and every third day, three-dimensional cell cultures were replenished with growth medium plus or minus 100pM TGFβ (R&D Systems, USA) or 10 μM TGFβ type I kinase inhibitor SB431542 (Sigma, Canada). For each experimental condition, ten differential interference contrast (DIC) micrographs were captured of 10 day-old live three-dimensional MDA-MB-231 cell-derived multicellular structures using light microscopy with a 20X objective (Olympus 1×70). Breast cancer cell-derived organoids were counted and scored as either non-invasive (smooth-surfaced spheroids) or invasive (disrupted structures with outward protrusions).

### Data and statistical analysis

For carrying out statistical analysis, all data were subjected to Student *t*-test or ANOVA followed by Student-Newman-Keuls test as a post-hoc test using InStat (Graphpad Software Inc., USA). Values of *p* ≤ 0.05 were considered statistically significant. All data were graphically represented as mean ± S.E.M. from experiments that were carried out at least three independent times. Statistical differences *p ≤ 0.05, **p ≤ 0.01 and ***p ≤ 0.001, unless otherwise stated.
